# Fulminant Myocarditis Secondary to Cardiac T-Cell Lymphoma

**DOI:** 10.1016/j.jaccas.2025.104743

**Published:** 2025-08-20

**Authors:** Yifei Wang, Huan Li, Shanquan Gao, Yuanwei Liu, Lihong Li, Zhiwei Han, Yilu Wang, Ping Zhang, Hongfang Yin, Rong He

**Affiliations:** a Department of Cardiology, Beijing Tsinghua Changgung Hospital, Tsinghua Medicine, Tsinghua University, Beijing, China; b Department of Pathology, Beijing Tsinghua Changgung Hospital, Tsinghua Medicine, Tsinghua University, Beijing, China; c Department of Hematology, Beijing Tsinghua Changgung Hospital, Tsinghua Medicine, Tsinghua University, Beijing, China

**Keywords:** cardiac lymphoma, mechanical circulatory support, multiorgan dysfunction, myocarditis, T-cell lymphoma

## Abstract

**Background:**

Cardiac T-cell lymphoma is a rare and aggressive malignancy that often presents with nonspecific symptoms and is difficult to diagnose early.

**Case Summary:**

A 73-year-old man presented with syncope, anorexia, and abdominal discomfort. During hospitalization, he developed hypereosinophilia and rapidly progressed to fulminant myocarditis. Despite steroid and immunoglobulin therapy, cardiac function declined, requiring extracorporeal membrane oxygenation and intra-aortic balloon pump support. Myocardial biopsy confirmed cardiac T-cell lymphoma with lymphocytic infiltration.

**Discussion:**

Cardiac T-cell lymphoma with fulminant myocarditis is exceptionally rare and carries a poor prognosis. Diagnosis is often delayed because of vague symptoms. This case highlights the value of early myocardial biopsy and histopathologic evaluation in rapidly deteriorating cardiac function.

**Take-Home Messages:**

Early myocardial biopsy is crucial in cases of unexplained myocarditis with rapid deterioration. Cardiac T-cell lymphoma is a rare and aggressive condition that can rapidly progress to fulminant myocarditis in certain cases, necessitating early diagnosis and intervention.

## Visual Summary

**Figure:**
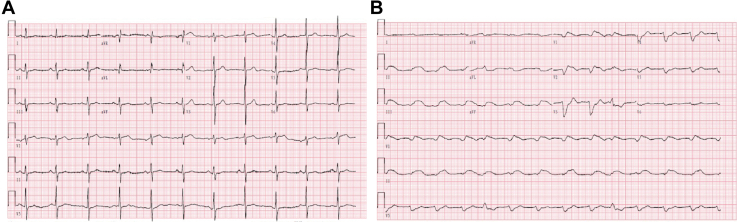

## History of Presentation

A 73-year-old man was admitted after a transient episode of syncope that occurred 6 days prior. The syncope was accompanied by dizziness, sweating, and nausea, but without chest pain, vomiting, fever, night sweats, or unintentional weight loss. Over the past 2 weeks, the patient had experienced a reduced appetite and intermittent abdominal pain. On admission, vital signs were stable, and physical examination revealed no significant abnormalities in cardiopulmonary or abdominal systems.

**Take-Home Messages**

• Early myocardial biopsy is crucial in cases of unexplained myocarditis with rapid deterioration.
• Cardiac T-cell lymphoma is a rare and aggressive condition that can rapidly progress to fulminant myocarditis in certain cases, necessitating early diagnosis and intervention.

## Past Medical History

The patient had no significant prior medical conditions.

## Differential Diagnosis

The initial differential diagnosis focused on common causes of syncope, including cardiogenic syncope, neurogenic syncope, and orthostatic hypotension.

## Investigations

**Figure 1:**
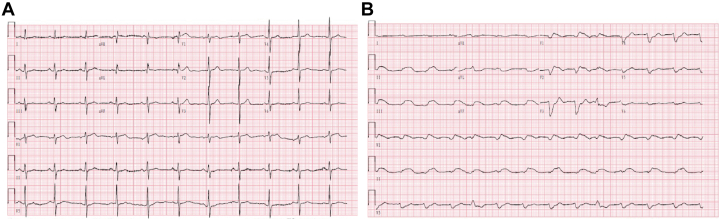
ECG Findings at Different Time Points After Admission (A) Day 0: Normal ECG with no significant abnormalities. (B) Day 16: High-degree atrioventricular block with visible ventricular escape rhythm and fusion beats. ECG = electrocardiography.

Laboratory tests on admission showed an elevated troponin level (0.0494 ng/mL; reference <0.014 ng/mL), normal white blood cell count (8.45 × 10^9^/L), and a normal creatinine level (94 μmol/L). Mild hypereosinophilia was noted, with an absolute eosinophil count of 0.95 × 10^9^/L (reference <0.5 × 10^9^/L) and a percentage of 11.3% (reference <5%). Electrocardiography revealed no significant abnormalities (Figure 1A). Echocardiography showed mild enlargement of the left atrium and ventricle, with a preserved left ventricular ejection fraction (LVEF) of 60% (Supplemental Table 1, Video 1). Coronary angiography demonstrated 50% to 70% stenosis in the posterior descending branch of the right coronary artery. Holter monitoring, brain magnetic resonance imaging, and orthostatic blood pressure measurements were unremarkable.

**Figure 2:**
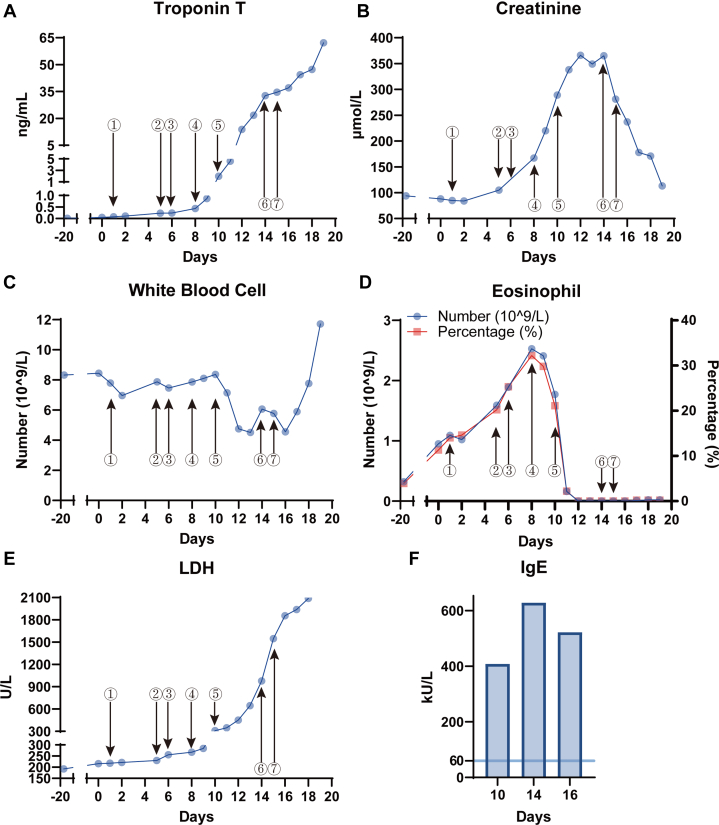
Trends in Laboratory Parameters at Different Time Points Before and After Admission Trends in laboratory parameters before and after admission: (A) troponin T concentration, (B) creatinine concentration, (C) white blood cell count, (D) eosinophil count and percentage, (E) lactate dehydrogenase (LDH) level, (F) IgE concentration (reference range <60 kU/L). Event ①: coronary angiography. Event ②: first occurrence of fever. Event ③: initiation of cefoperazone-sulbactam for anti-infective therapy. Event ④: discontinuation of cefoperazone-sulbactam. Event ⑤: initiation of intravenous methylprednisolone and immunoglobulin therapy. Event ⑥: initiation of extracorporeal membrane oxygenation and intra-aortic balloon pump support. Event ⑦: initiation of intravenous dexamethasone therapy.

**Figure 3:**
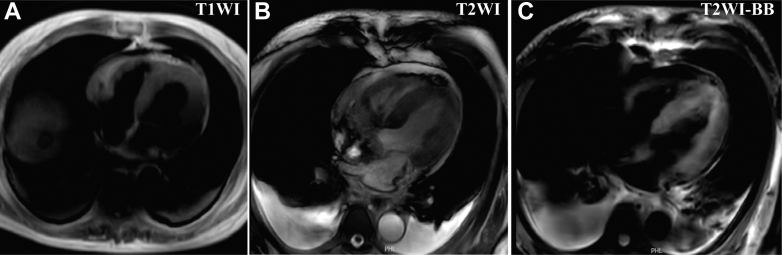
Cardiac Magnetic Resonance Findings Cardiac magnetic resonance performed on day 12 post-admission: (A) T1-weighted imaging (T1WI), (B) T2WI, (C) T2-weighted black blood imaging (T2WI-BB). The images demonstrated pericardial effusion and heterogeneous myocardial thickening. The T2WI-BB sequence reveals increased myocardial signal intensity predominantly in the apical region of the left ventricle.

However, the patient's condition deteriorated with a continuous rise in troponin levels (Figure 2A). On day 5, a fever of 37.9 °C was observed, accompanied by elevated troponin, creatinine, and eosinophil levels, whereas the white blood cell count remained stable (Figure 2, Supplemental Figure 1). Despite initial treatment with antibiotics and methylprednisolone, eosinophil levels decreased, but cardiac biomarkers continued to rise. As the condition progressed, serial echocardiography revealed the development of significant myocardial edema, new pericardial effusion, and a gradual decline in cardiac function (Supplemental Table 1, Video 2). On day 12, cardiac magnetic resonance (Figure 3) demonstrated diffuse myocardial edema predominantly involving the left ventricular apex, and the LVEF measured from short-axis cine images was 46% (Video 3). Because of worsening cardiac function, extracorporeal membrane oxygenation (ECMO) and intra-aortic balloon pump were implemented. A diagnosis of fulminant myocarditis was established based on myocardial injury and unstable hemodynamics. Blood test results for autoimmune markers, viruses, and cultures were negative, though IgE levels were markedly elevated (Figure 2).

**Figure 4:**
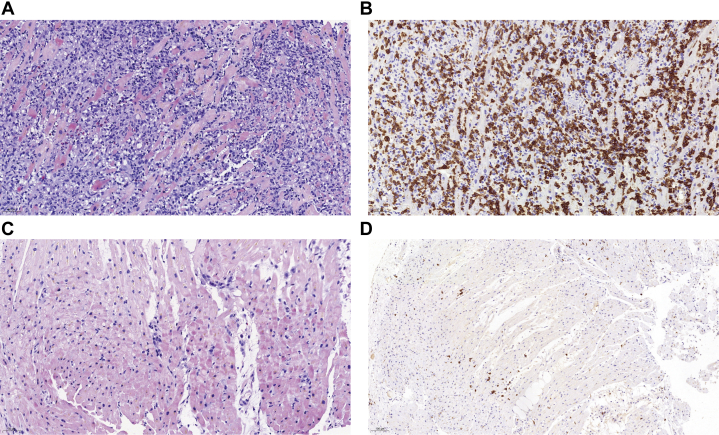
Histopathology and Immunohistochemistry of Myocardial Biopsy (A) Basal myocardial biopsy (HE, ×20 magnification) showing diffuse proliferation of atypical lymphocytes with irregular nuclear contours, accompanied by a small amount of eosinophilic and histiocytic infiltration. (B) Immunohistochemistry of basal myocardial biopsy showing CD3-positive tumor cells (×20 magnification). (C) Apical myocardial biopsy (HE, ×20 magnification) showing small lymphocytic infiltration, cardiomyocyte atrophy, and lipofuscin deposition. (D) Immunohistochemistry of apical myocardial biopsy showing CD3-positive lymphocytes (×10 magnification). HE = hematoxylin and eosin.

A myocardial biopsy on day 13, sampling the interventricular septum of the right ventricle, revealed no microbial DNA via next-generation sequencing. Biopsy of the basal myocardium showed disruption of normal myocardial architecture with infiltration by numerous lymphoid tumor cells (Figure 4A). Immunohistochemical analysis demonstrated strong positivity for CD3 (Figure 4B), as well as CD4, CD5, and CD8, with partial positivity for CD30, granzyme B, and T-cell intracellular antigen-1 (Supplemental Figure 2). The Ki-67 proliferation index was 85%, and T-cell receptor gene rearrangement testing confirmed monoclonal rearrangement (Supplemental Figure 3). Apical myocardial biopsy revealed cardiomyocyte degeneration, atrophy, and lipofuscin deposition, with a small number of CD3-positive lymphocytes present (Figures 4C and 4D). These findings, along with the immunohistochemical markers, confirmed the diagnosis of T-cell lymphoma with lymphocytic myocarditis.

## Management

As shown in Figure 2, after the onset of fever, the patient was treated with cefoperazone-sulbactam. However, as his condition continued to deteriorate, he was transferred to the cardiac intensive care unit on day 10. He received escalating doses of methylprednisolone (increasing from 20 mg to 200 mg daily) and intravenous immunoglobulin (20 g daily for 3 days), which resulted in a reduction in eosinophil levels and partial improvement in renal function. Despite these interventions, the patient's cardiac function continued to worsen, leading to the initiation of ECMO and intra-aortic balloon pump. After biopsy results confirmed the diagnosis of cardiac T-cell lymphoma with lymphocytic myocarditis, his treatment regimen was adjusted to dexamethasone (20 mg daily). However, further chemotherapy was not pursued because of the patient's inability to tolerate it, as the chemotherapy would likely exacerbate the extensive local lymphoma destruction, intensify inflammation, and accelerate myocarditis progression.

## Outcome and Follow-Up

Despite aggressive treatment, the patient's condition continued to deteriorate. He became comatose, with severe atrioventricular block, widespread inflammation, and only 15% LVEF remaining on day 16. After discussing the potential benefits of positron emission tomography–computed tomography, bone marrow aspiration, and lymph node biopsy with the family, they opted for palliative care because of the patient's fragile state. Ultimately, his condition worsened further, and despite ECMO support, his vital signs became unsustainable, leading to clinical death on the 19th day of hospitalization.

## Discussion

This case presents a rare instance of cardiac T-cell lymphoma, diagnosed through myocardial biopsy, accompanied by lymphocytic myocarditis and multiorgan involvement. The primary challenge was the patient's nonspecific symptoms, which rapidly progressed to a fatal outcome, impacting the heart, kidneys, and hematological systems. The concealed onset, ambiguous symptoms, rapid progression, and poor prognosis made the diagnosis particularly difficult.

The most significant threat to the patient was fulminant myocarditis. According to guidelines from the European and Chinese Societies of Cardiology,^1^^,^^2^ elevated cardiac injury markers, abnormal imaging findings, and unstable hemodynamics confirmed the diagnosis of fulminant myocarditis, which carries a 30-day mortality rate of 22%.^3^ The most common cause of fulminant myocarditis is an overactive inflammatory response, often secondary to viral infection. The standard treatment includes life-support therapies along with immunomodulatory treatments.^2^ Endomyocardial biopsy remains the gold standard for diagnosing myocarditis, especially when initial treatments fail and a pathologic diagnosis is urgently needed.^1^^,^^4^ In this case, the biopsy revealed cardiac T-cell lymphoma and lymphocytic myocarditis, underscoring its critical role in confirming the diagnosis. Eosinophilic myocarditis secondary to T-cell lymphoma has also been reported to cause syncope, as reported in a similar case involving myocardial injury.^5^ The myocardial biopsy, performed after several days of corticosteroid treatment, revealed only a few eosinophils, making it unclear whether eosinophilic myocarditis was present at the onset.

Notably, the earliest prominent abnormality on admission was hypereosinophilia. Although this raised suspicion for hypereosinophilic syndrome, the diagnostic criteria were not fulfilled, which requires either persistent eosinophilia on at least 2 occasions 1 month apart, or histologic evidence of extensive eosinophilic infiltration in tissues. Therefore, we focused mainly on the differential diagnosis of hypereosinophilia. Given the patient's coronary angiography examination and antibiotics treatments, drug reaction with eosinophilia and systemic symptoms was considered. However, the absence of a rash and the failure of eosinophil reduction to stop myocardial injury ruled out drug reaction with eosinophilia and systemic symptoms. Infection- and autoimmune-related causes were ruled out based on negative cultures, serologies, and next-generation sequencing. T-cell lymphoma remains a rare but important cause of secondary hypereosinophilia, typically mediated by cytokines such as interleukin-5, which stimulate eosinophil proliferation and activation.^6^ In this case, the hypereosinophilia was likely a paraneoplastic manifestation of the underlying aggressive lymphoma.

The median survival time for cardiac T-cell lymphoma is typically only 2 months,^7^ and the presence of fulminant myocarditis further worsens the prognosis. In this case, conventional treatments such as corticosteroids and immunoglobulin were ineffective in halting the overactive myocardial inflammation due to the ongoing lymphoma proliferation and lymphocyte recruitment. After these initial therapies, a multidisciplinary team discussion was held to evaluate the potential use of second-line immunosuppressive agents. Although such agents might assist in modulating local myocardial inflammation, it was considered that the primary threat to the patient's survival was the uncontrolled proliferation of T-cell lymphoma. Without directly targeting the tumor burden, additional immunosuppressive therapy was unlikely to alter the disease trajectory and could significantly increase the risk of infection. In most lymphoma-related myocarditis cases, chemotherapy, particularly cyclophosphamide, hydroxydaunorubicin, oncovin, and prednisone for T-cell lymphoma, is the primary treatment.^8^ However, when complications arise or lymphoma becomes resistant to chemotherapy, treatment options become limited, and conservative therapies are often adopted.^9^ Dexamethasone was selected because of its dual role in suppressing inflammation and exerting antilymphoma effects through induction of T-cell apoptosis.

There were several limitations in the diagnosis of this case. Positron emission tomography–computed tomography, lymph node biopsy, bone marrow aspiration, and genetic testing are critical for confirming the stages of lymphoma, determining its pathologic type, and distinguishing the etiology of reactive eosinophilia. However, because of the rapid disease progression and initially unclear etiology, by the time the diagnosis of T-cell lymphoma was established, the patient's condition had deteriorated to the point where further invasive examinations were no longer feasible. This delay made it difficult to determine the lymphoma's stage and whether it was primary or secondary cardiac lymphoma. In addition, contrast-enhanced cardiac magnetic resonance was not performed because of the patient's intolerance, and global longitudinal strain was not assessed because bedside echocardiography in the cardiac intensive care unit lacked electrocardiography synchronization. These limitations restricted the evaluation of myocardial inflammation and function. In retrospect, an earlier diagnosis might have allowed for more aggressive treatment and potentially offered the patient a chance at a better outcome. The unexplained hypereosinophilia seen initially could have been a prompt for more targeted diagnostic investigations, which might have facilitated a more decisive diagnosis and intervention.

## Conclusions

This case illustrates a rare presentation of fulminant myocarditis secondary to cardiac T-cell lymphoma and highlights the importance of early myocardial biopsy and targeted intervention in patients with unexplained fulminant myocarditis.

## Funding Support and Author Disclosures

The authors have reported that they have no relationships relevant to the contents of this paper to disclose.

## Appendix

Video 1 Transthoracic Echocardiography on Day 0

Video 2 Transthoracic Echocardiography on Day 16

Video 3 Cardiac Magnetic Resonance Cine Imaging (Short-Axis View) Short-axis cine sequence from cardiac magnetic resonance on day 12 showing decreased global contractility of the left ventricle, with an estimated ejection fraction of 46%.
