# Supplementary material for: Fulminant Myocarditis Secondary to Cardiac T-Cell Lymphoma
Source: JACC Case Rep. 2025 Aug 20;30(24):104743. doi: 10.1016/j.jaccas.2025.104743 (PMC12371419; doi:10.1016/j.jaccas.2025.104743)
Supplement: Supplemental Materials [file mmc4.docx]

**

**

**Supplementary Figure 1. Trends in Laboratory Parameters at Different Time Points Before and After Admission**

Trends in laboratory parameters before and after admission: (A) Neutrophil count, (B) Lymphocyte count, (C) Monocyte count, (D) Total Bilirubin level, (E) Alanine Aminotransferase level, (F) Aspartate Aminotransferase level, (G) Potassium level and (H) Sodium level. Event ①: Coronary angiography. Event ②: First occurrence of fever. Event ③: Initiation of Cefoperazone-Sulbactam for anti-infective therapy. Event ④: Discontinuation of Cefoperazone-Sulbactam. Event ⑤: Initiation of intravenous methylprednisolone and immunoglobulin therapy. Event ⑥: Initiation of ECMO and IABP support. Event ⑦: Initiation of intravenous dexamethasone therapy.

**Supplementary Figure 2. Immunohistochemistry of** **Myocardial Biopsy**

Biopsy from the basal region of the heart (20x magnification), the immunohistochemistry of CD4 (A), CD5 (B), CD8 (C), CD30 (D), granzyme B (E) and TIA-1 (F) were shown.

**

**

**Supplementary Figure 3. T cell receptor (TCR) gene rearrangement**

TCR gene rearrangement testing showed monoclonal rearrangement of TCRB (TCR-β). (A)TCRB-A(Vβ+Jβ1/2). (B)TCRB-B(Vβ+Jβ2).

|  | Day 0 | Day 10 | Day 11 | Day 13 | Day 15 | Day 16 |
| --- | --- | --- | --- | --- | --- | --- |
| LVEF | 60% | 56% | 56% | 55% | 20% | 15% |
| E (cm/s) | 63 | 54 | 49 | 108 | 81 | 52 |
| A (cm/s) | 103 | 98 | 87 | 81 | 59 | 20 |
| E/A | 0.6 | 0.6 | 0.6 | 1.3 | 1.4 | 2.6 |
| E/e’ | 9.7 | 11.7 | 7.5 | 15.4 | 27.0 | 20.8 |
| IVS (mm) | 10 | 8 | 14 | 14 | 15 | 15 |
| LAAPD (mm) | 40 | 37 | 37 | 33 | 38 | 38 |
| LVEDD (mm) | 58 | 60 | 59 | 52 | 62 | 56 |
| LVESD (mm) | 40 | 42 | 34 | 31 | 48 | 48 |
| RVAPD (mm) | 23 | 25 | 23 | 22 | 22 | 23 |
| PE Depth (mm) | - | - | 3 | 3 | 6 | 5 |

**Supplementary Table 1. Echocardiographic data at different time point after admission**

Abbreviations: Left Ventricular Ejection Fraction (LVEF), Mitral Valve E-wave Velocity (E), Mitral Valve A-wave Velocity (A), E/A Ratio (E/A), E/e’ Ratio (E/e’), Interventricular Septum Thickness (IVS), Left Atrial Anteroposterior Diameter (LA APD), Left Ventricular End-Diastolic Diameter (LVEDD), Left Ventricular End-Systolic Diameter (LVESD), Right Ventricular Anteroposterior Diameter (RVAPD), Pericardial Effusion Depth at the right ventricular free wall (PE Depth).
